# Optimization on Preparation Condition of Propolis Flavonoids Liposome by Response Surface Methodology and Research of Its Immunoenhancement Activity

**DOI:** 10.1155/2013/505703

**Published:** 2013-02-28

**Authors:** Ju Yuan, Yu Lu, Saifuding Abula, Yuanliang Hu, Jiaguo Liu, Yunpeng Fan, Xiaojuan Zhao, Deyun Wang, Xu Liu, Cui Liu

**Affiliations:** ^1^Institute of Traditional Chinese Veterinary Medicine, College of Veterinary Medicine, Nanjing Agricultural University, Nanjing 210095, China; ^2^National Research Center of Veterinary Biologicals Engineering and Technology, Jiangsu Academy of Agricultural Science, Nanjing 210014, China; ^3^College of Veterinary Medicine, Xinjiang Agricultural University, Urumchi 830052, China

## Abstract

The aim of this study is to prepare propolis flavonoids liposome (PFL) and optimize the preparation condition and to investigate further whether liposome could promote the immunoenhancement activity of propolis flavonoids (PF). PFL was prepared with ethanol injection method, and the preparation conditions of PFL were optimized with response surface methodology (RSM). Moreover, the immunoenhancement activity of PFL and PF *in vitro* was determined. The result showed that the optimal preparation conditions for PFL by response surface methodology were as follows: ratio of lipid to drug (w/w) 9.6 : 1, ratio of soybean phospholipid to cholesterol (w/w) 8.5 : 1, and speed of injection 0.8 mL*·*min^−1^. Under these conditions, the experimental encapsulation efficiency of PFL was 91.67 ± 0.21%, which was close to the predicted value. Therefore, the optimized preparation condition is very reliable. Moreover, the results indicated that PFL could not only significantly promote lymphocytes proliferation singly or synergistically with PHA, but also increase expression level of IL-2 and IFN-*γ* mRNA. These indicated that liposome could significantly improve the immunoenhancement activity of PF. PFL demonstrates the significant immunoenhancement activity, which provides the theoretical basis for the further experiment *in vivo*.

## 1. Introduction

Natural products are a promising source for the discovery of new pharmaceuticals. In the last decades, several works dealing with propolis composition and biological properties have been published, revealing the interest of researchers on this bee product and its potential for the development of new drugs as well [1–4]. Propolis has been employed extensively since ancient times in Egypt, Greece, Roman Empire, and so on. Its use continues today as a popular remedy and is available in either in pure form or combined with other natural products in cosmetics and as a constituent of healthy foods. 

Propolis presents plenty of biological and pharmacological properties, such as immunomodulatory, antitumor, anti-inflammatory, antioxidant, antibacterial, antiviral, antifungal, and antiparasite activities, among others [5–10]. Propolis mechanisms of action have been widely investigated in the last years, using different experimental models *in vitro* and *in vivo*. Researchers have been interested in the investigation of isolated compounds responsible for propolis action and find flavonoids are one of the most important groups [11]. Propolis flavonoids (PF) are responsible for many of its biological and pharmacological activities [12]. Although PFs have good effect as antioxidant, antitumor, immunomodulatory, and so forth, they are easy to be oxidized [13], which makes the flavonoids themselves be not stable. The storage, use, and function of propolis were affected with its instability. 

Liposome is a synthetic bilayer membrane vesicle with phosphorus and has good affinity on cell membrane. For their biodegradability, biocompatibility, low toxicity, and their ability to entrap both lipophilic and hydrophilic drugs [14], liposomes are known for their potential and actual uses in targeted drug delivery. If PFs are encapsulated with liposome, their stability will be increasingly promoted. However, the effective factors on preparation of propolis flavonoids liposome (PFL) are various, and the preparation conditions need to be optimized. In additional, it is also important whether the biological activity is changed with liposome encapsulation. 

Response surface methodology (RSM) is a collection of mathematical and statistical techniques useful for analyzing the effects of several independent variables [15–17]. In many processes, the relationship between the response and the independent variables is usually unknown; therefore the first step in RSM is to approximate the function (response) in terms of analyzing variables (independent variables). Usually, this process employs a low-order polynomial equation in a predetermined region of the independent variables, which is later analyzed to locate the optimum values of independent variables for the best response [17]. 

Therefore, in the present study, RSM was employed to optimize the preparation conditions of PF, and the biological activity of PF was compared between preencapsulation and postencapsulation. The aim of this strategy is to optimize the best preparation condition of PFL and observe immunoenhancement activity *in vitro*, which provide the theoretical basis for the further experiment *in vivo* to study whether PFL could promote immune response.

## 2. Materials and Methods

### 2.1. Materials

Propolis was purchased from Dahua Chinese Traditional Medicine Company in Nanjing, Jiangsu Province. PFs were prepared in our laboratory (briefly, propolis was extracted with 95% ethanol for three times, and the ethanol solution was retrieved. Then, the precipitation was extracted with ethyl acetate for three times, and then the ethyl acetate was retrieved. Finally, the precipitation was dried in vacuum, and PF was obtained.). Soybean phospholipid (number 20110908) was manufactured by Shanghai Taiwei Pharmaceutical Co., Ltd, and Cholesterol (number 20110706) purchased from Anhui Tianqi Chemical Technology Co., Ltd. Protamine (Sigma, P4380) was dissolved by physiological saline to 10 mg mL^−1^. Lymphocyte separation medium (number 110326) was manufactured by Shanghai Huajing Biology Inc. RPMI-1640 (GIBCO) with the supplement of 100 IU mL^−1^ benzylpenicillin, 100 IU mL^−1^ streptomycin, and 10% fetal bovine serum was used for washing and resuspending cells, diluting mitogen, and culturing the cells. Phytohemagglutinin (PHA, Sigma, number L-8754), as a T-cell mitogen, was dissolved into 0.1 mg mL^−1^ with RPMI-1640. Hanks' solution was used for diluting blood. The 3-(4,5-dimethylthiazol-2-yl)-2,5-diphenyltetrazolium bromide (MTT, American Co.) was dissolved into 5 mg mL^−1^ with calcium and magnesium-free (CMF) phosphate-buffered saline (PBS, pH 7.2). These reagents were filtered through a 0.22 *μ*m millipore membrane filter. PHA solution was stored at −20°C, MTT solution at 4°C in dark bottles, and RPMI-1640 at 4°C. Dimethyl sulfoxide (DMSO, number 20090519) was produced by Kemou Institute of Chemical Engineering in Tianjing, 5× PrimeScript RT Master Mix, SYBR Premix Ex Taq, ROX Reference Dye (50x), Takara Inc.

### 2.2. Preparation of PF Liposome

Propolis flavonoids (PF) liposome was prepared with ethanol injection method [18]. Lecithin, cholesterol, and propolis flavonoids were dissolved in about 10 mL of ethanol, and the ethanol was injected into the buffer (40°C, PBS) with a slow speed and continued to thermostatic mixing. Liposomes formed spontaneously after further evaporation of the residual ethanol. The resulting mixture was homogenized with ultrasonication for 30 min to form the small single-chamber liposome (Ultrasonic Cleaner KQ5200B, Kunshan Sonicatic equipment Inc. China) [18]. Ultimately, the solution was filtered with 0.8 *μ*m, 0.45 *μ*m, and 0.22 *μ*m millipore membrane successively [19].

#### 2.2.1. Entrapment Efficiency (EE) of PFL Array

0.8 mL of PFL was added in 10 mL centrifuge tube and mixed with 0.8 mL of protamine solution (10 mg·mL^−1^). After 3 min, 3.0 mL of physiological saline was added. After adequate mixing, this suspension was centrifuged at 3000× r·min^−1^ at the room temperature for 30 min. All supernatant was taken from centrifuge tube, setting the volume to 10 mL with physiological saline, 5 mL was taken to assay the content of PF by ultraviolet spectrophotometry method with Rutin standard substance [20], and the content of PF was called the content of free drug. The precipitation remaining in centrifuge tube was dissolved by 3.0 mL of Triton X-100 and then setting the volume to 10 mL with physiological saline. Five millilitres of the solution were taken to assay the content of PF by Rutin standard method and called the content of encapsulated drug. The formula to calculate liposome encapsulation efficiency was EE% = (1 − *C*_*f*_/*C*_*t*_), *C*_*f*_: the content of free drug, *C*_*t*_: the total content of drug [21–23].

#### 2.2.2. Optimization of PFL Preparation

Based on the single-factor test, three factors, ratio of lipid to drug w/w (A), ratio of soybean phospholipid to cholesterol w/w (B), and speed of injection/mL·min^−1^ (C), were selected to optimize the preparation conditions of PF liposome. The selected factors were subjected to response surface methodology (RSM) with a three-factor three-coded level Box-Behnken design (BBD) to optimize the preparation conditions of PFL. The range and the levels of experimental variables investigated in this study are presented in Table 1. In addition, several verification experiments were done according to the optimal conditions. 

### 2.3. Immunoenhancement Activity of PFL *In Vitro*

#### 2.3.1. T Lymphocyte Proliferation Assay

Blood samples were collected from nonimmunized White Roman chickens at 60 days old (provided by Tangquan Poultry Farm), transferred immediately into aseptic capped tubes containing sodium heparin, then diluted with an equal volume of Hanks' solution, and carefully layered on the surface of lymphocyte separation medium. After centrifugation at 800 ×g for 15 min, the lymphocytes were collected and washed twice with Hanks' solution. The resulting pellet was resuspended and diluted to 2.5 × 10^6^ mL^−1^ with RPMI-1640 with fetal bovine serum after the cell viability was assessed by trypan blue exclusion. The solution was divided into two parts: one part was added with PHA, and the other part was directly added. They were, respectively, incubated into 96-well culture plates, 100 *μ*L per well. Then, PFL, PF, and BL at series of concentrations were added, in cell control group and PHA control group, RPMI-1640 medium and PHA, respectively, 100 *μ*L per well, four wells each concentration. The final concentration of PHA reached to 20 *μ*g mL^−1^. The plates were, respectively, incubated in a humid atmosphere with 5% CO_2_ (Revco, Co., USA) at 39.5°C for 48 h. Briefly, 30 *μ*L of MTT (5 mg mL^−1^) was added into each well at 4 h before the end of incubation. Then the plates were centrifuged at 1000 ×g for 10 min at room temperature. The supernatant was removed carefully, and 100 *μ*L of DMSO was added into each well. The plates were shaken for 5 min to dissolve the crystals completely. The absorbance of cells in each well was measured by microliter enzyme-linked immunosorbent assay reader (Model RT-6000, Leidu Co., Ltd. Shenzhen City) at a wavelength of 570 nm (*A*_570_ value) as the index of lymphocytes proliferation [24].

#### 2.3.2. The Experiment of Gene Expression

*Preparation of Lymphocyte. *Blood samples were collected from nonimmunized White Roman chickens at 60 days old (provided by Tangquan Poultry Farm), transferred immediately into aseptic capped tubes containing sodium heparin, then diluted with an equal volume of Hanks' solution, and carefully layered on the surface of lymphocyte separation medium. After centrifugation at 800 ×g for 15 min, the lymphocytes were collected and washed twice with Hanks' solution. Cell viability was assessed by trypan blue exclusion. The resulting pellet was resuspended and diluted to 1.0 × 10^7^ mL^−1^ with RPMI-1640 with fetal bovine serum and incubated in 24-well culture plates with 0.8 mL/well, then different concentrations PFL and PF were added into, and each sample seeded four wells, 1.0 mL per well. PHA with a working concentration 20 *μ*g/mL was used for the purification of lymphocytes. The final volume of each well reached to 2.0 mL. The lymphocytes were collected to isolate the RNA to amplify IL-2 and IFN-*γ* after the plates were incubated at 5% CO_2_ at 39.5°C for 36 h.

*Total RNA Isolation.* Total RNA was isolated from peripheral T lymphocyte by using Trizol Reagent (Invitrogen) following the illustration provided by the manufacturer. The quality of isolated RNA was determined by ultraviolet spectrophotometry, and the optical density was 1.8–2.0.

*Reverse Transcription (RT)*. RT was performed according to literature [25]. Briefly, the system of reverse transcription was 10 *μ*L, containing 400 ng of total RNA, 2 *μ*L 5× PrimeScript RT Master Mix∗ (for real time), and adding RNase Free dH_2_O until 10 *μ*L at 37°C for 15 min, 85°C for 5 second, and 4°C for random period of time. First strand cDNAs were store at −20°C until further use.

*PCR Amplification.* Amplification was carried out in a total volume of 20 *μ*L. Each PCR reaction included 6.8 *μ*L of dH_2_O, 10.0 *μ*L of SYBR Premix Ex Taq (2×), 0.4 *μ*L of PCR Forward Primer, 0.4 *μ*L of PCR Reverse Primer, 0.4 *μ*L of ROX Reference Dye (50×), and 2 *μ*L of cDNA. PCR cycling conditions included a 95°C heating step for 30 s at the beginning of every run. The tubes were then cycled at 95°C for 5 s, annealed at 60°C for 31 s, and extend at 72°C for 1 min, 40 cycles. A melting curve was generated at the end of every run to ensure product uniformity [26]. The specific primer sequences used were as follows:

 IL-2 (162 bp):
   5′-CTTTGGCTGTATTTCGG-3′,
   5′-CTGGGTCTCAGTTGGTG-3′;
 IFN-*γ* (171 bp):
   5′-GCTGACGGTGGACCTATT-3′,
   5′-TCCTCTGAGACTGGCTCCTT-3′;
 
*β*-action (280 bp):
   5′-ACGTCGCACTGGATTTCG-3′,
   5′-TGTCAGCAATGCCAGGGT-3′.

Analysis of relative gene expression data is referring to the literature [27].

### 2.4. Statistical Analysis

Data of optimization of PFL preparation are analyzed by Design-Expert 7.0 software (Stat-Ease, Inc), second-order polynomial equation, and ANOVA of the quadratic regression model, and the optimal conditions were showed. Data of immunoenhancement activity experiment are expressed as mean ± standard errors (S.E.). Duncan, LSD's multiple range tests and *t*-tests were used to determine the difference among groups. *P* values of less than 0.05 were considered to be statistically significant. 

## 3. Results

### 3.1. Statistical Analysis and the Model Fitting of PFL Preparation Optimization

There were 17 experimental runs for optimizing the three individual parameters in the Box-Behnken design (BBD), and the experimental conditions and the EE of PFL according to the factorial design were shown in Table 2. The results showed that the maximum EE value (92.60%) was found in conditions of *X*_1_ = 10 : 1, *X*_2_ = 8 : 1, and *X*_3_ = 0.6 mL·min^−1^. The values of regression coefficients were calculated, and the response variable and the test variables were related by the following second-order polynomial equation:

(1) EE=91.30−0.91X1+2.21X2−0.029X3−0.64X1X2−2.36X1X3+0.36X2X3−7.32X12−4.87X22−7.57X32.

The statistical significance of the regression model was checked by *F*-test and *P* value, and the analysis of variance (ANOVA) for the response surface quadratic model was shown in Table 3. The determination coefficient (*R* ^2^ = 0.9898), showed by ANOVA of the quadratic regression model, indicating that the model was highly significant and adequate for prediction within the range of experimental variables. The *P* value was used as a tool to check the significance of each coefficient, and the smaller the *P* value was, the more significant the corresponding coefficient was. In this table the linear coefficients (*X*_1_, *X*_2_), a quadratic term coefficient (*X*_1_^2^, *X*_2_^2^, and *X*_3_^2^), and the interaction coefficient (*X*_1_ × *X*_3_) were found significantly (*P* < 0.05). The other term coefficients were not significant (*P* > 0.05). By the Design-Expert software to further optimize the preparation conditions, the optimum for preparation of PFL conditions obtained was as follows: ratio of lipid to drug (w/w) 9.6 : 1, ratio of soybean phospholipid to cholesterol (w/w) 8.5 : 1, and speed of injection 0.8 mL·min^−1^. 

### 3.2. Verification of Predictive Mode

The suitable of the model equation for predicting the optimum response value was tested by using the selected optimal conditions. The maximum predicted and experimental value of EE was given in Table 4. To ensure the predicted result was not biased toward the practical value, experiment rechecking was performed by these modified optimal conditions: ratio of lipid to drug (w/w) of 9.6 : 1, ratio of soybean phospholipid to cholesterol (w/w) of 8.5 : 1, and speed of injection 0.8 mL·min^−1^. A mean value of 91.67 ± 0.21% (*n* = 3) obtained from real experiments, demonstrated the validation of the RSM model, indicating that the model was adequate for the preparation process.

### 3.3. Effect of PFL on T Lymphocyte Proliferation *In Vitro*

#### 3.3.1. Effect of T Lymphocyte Proliferation in Single Stimulation of Drugs

**Figure 1:**
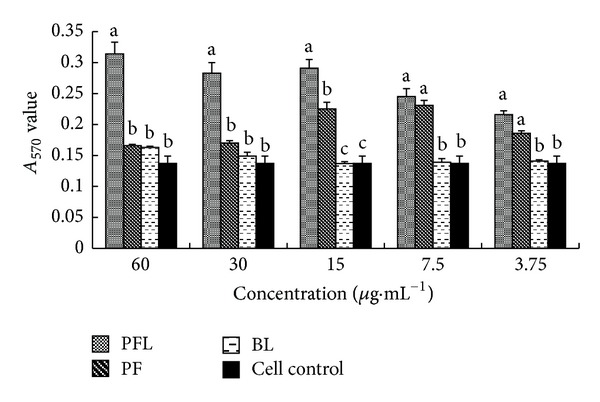
Changes of T lymphocyte proliferation in single stimulation with drugs (*A*_570_ values). ^a–c^Bars in the same day without the same superscripts differ significantly (*P* < 0.05).

The results are listed in Figure 1. At 60–15 *μ*g·mL^−1^, the *A*_570_ values of PFL group were the highest in those four groups and significantly higher than those of PF, BL, and cell control groups (*P* < 0.05). At 15–3.75 *μ*g·mL^−1^, the *A*_570_ values of PF groups were significantly higher than those in BL and cell control group (*P* < 0.05). 

#### 3.3.2. Effect of T Lymphocyte Proliferation in Synergistical Stimulation of Drugs with PHA

**Figure 2:**
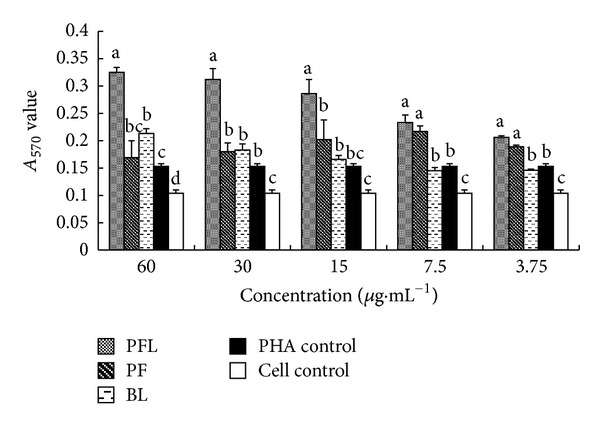
Changes of T lymphocyte proliferation in synergistical stimulation of drugs with PHA (*A*_570_ values). ^a–d^Bars in the same day without the same superscripts differ significantly (*P* < 0.05).

The results are shown in Figure 2. At 60–3.75 *μ*g·mL^−1^, the *A*_570_ values of PFL group were the highest in those five groups. At 60–15 *μ*g·mL^−1^, the *A*_570_ values of PFL group were significantly higher than those in PF, BL, PHA, and cell control groups (*P* < 0.05). At 7.5–3.75 *μ*g·mL^−1^, the *A*_570_ values of PFL and PF groups were significantly higher than those in BL, PHA, and cell control groups (*P* < 0.05).

#### 3.3.3. Effect of PFL on Expression Level of IL-2 mRNA

**Figure 3:**
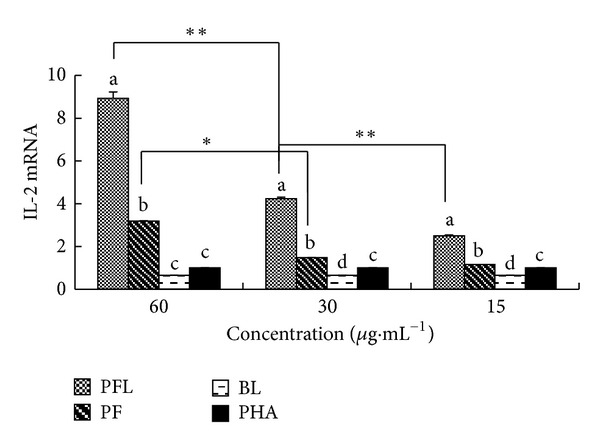
Effect of PFL on expression level of IL-2 mRNA. ^ a–d^Bars in the same day without the same superscripts differ significantly (*P* < 0.05). *means significantly different between two groups (*P* < 0.05). **means significantly different between two groups (*P* < 0.01).

The results are listed in Figure 3. IL-2 mRNA level of PFL and PF groups at 60–15 *μ*g·mL^−1^ were significantly higher than those of BL and PHA groups (*P* < 0.05), and these of PFL group being significantly higher than those of PF groups (*P* < 0.05). IL-2 mRNA level of PFL group at 60 *μ*g·mL^−1^ was significantly higher than those of PFL at 30 *μ*g·mL^−1^ and 15 *μ*g·mL^−1^ groups, and at 30 *μ*g·mL^−1^ being significantly higher than at 15 *μ*g·mL^−1^ group. 

#### 3.3.4. Effect of PFL on Expression Level of IFN-*γ* mRNA

**Figure 4:**
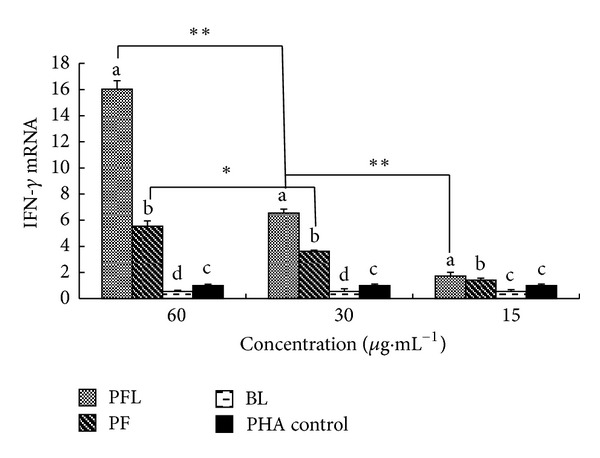
Effect of PFL on expression level of IFN-*γ* mRNA. ^a–d^Bars in the same day without the same superscripts differ significantly (*P* < 0.05). *means significantly different between two groups (*P* < 0.05). **means significantly different between two groups (*P* < 0.01).

The results are showed in Figure 4. IFN-*γ* mRNA level of PFL and PF groups at 60–15 *μ*g·mL^−1^ were significantly higher than those of BL and PHA groups (*P* < 0.05) and these of PFL group being significantly higher than those of PF groups (*P* < 0.05). IFN-*γ* mRNA level of PFL group at 60 *μ*g·mL^−1^ was significantly higher than those of PFL at 30 *μ*g·mL^−1^ and 15 *μ*g·mL^−1^ groups, and at 30 *μ*g*·*mL^−1^ being significantly higher than at 15 *μ*g·mL^−1^ group. 

## 4. Discussion

Encapsulation efficiency is critical factor to appraise the quantity of liposome drugs and is affected with many factors. In order to achieve high encapsulation efficiency of PFL, in the experiment the preparation conditions of PFL must be optimized. 

Traditionally, optimization in analytical chemistry has been carried out by monitoring the influence of one factor at a time on an experimental response. Its major disadvantage is that it does not include the interactive effects among the variables studied. As a consequence, this technique does not depict the complete effects of the parameter on the response [28, 29]. Another disadvantage of the one-factor optimization is the increase in the number of experiments necessary to conduct the research, which leads to an increase of time and expenses as well as an increase in the consumption of reagents and materials. Response surface methodology is a designed regression analysis meant to predict the value of a dependent variable based on the controlled values of the independent variables [30, 31]. The use of RSM in the process optimization stage leads to the need for an experimental design, which can generate a lot of samples for consumer evaluation in a short period of time, and thus laboratory level tests are more efficient [30]. The product optimization time is greatly reduced from traditional “cook and look” optimization techniques [29]. From the parameter estimates, it can be determined which variable contributes the most to the prediction model, thereby allowing the product researcher to focus on the variables that are most important to the product acceptance [32]. 

Therefore, in the study, preparation conditions of PFL must be optimized with response surface methodology (RSM). The optimization of PFL was ratio of lipid to drug (w/w) of 9.6 : 1, ratio of soybean phospholipid to cholesterol (w/w) of 8.5 : 1, speed of injection 0.8 mL*·*min^−1^, and mean encapsulation efficiency of 91.67 ± 0.21%. It was similar to the predicted value 91.59%, which indicated that the model was adequate for the preparation process.

*In vitro*, the immunomodulatory activity was compared between PFL and PF. The results showed that whether in single stimulation or in synergistical stimulation with PHA, the *A*_570_ values of PFL group in certain concentrations (15–60 *μ*g·mL^−1^) were significantly higher than those of PF group (Figures 1 and 2). Furthermore, the results suggested that at concentrations of 15–60 *μ*g·mL^−1^, the expressions of IL-2 mRNA and IFN-*γ* mRNA in PFL group were significantly higher than those in PF group (Figure 3 and Figure 4); in addition, the increases of expressions of IL-2 mRNA and IFN-*γ* mRNA in PFL group were significant from 15 *μ*g·mL^−1^ to 60 *μ*g·mL^−1^. These indicated that the immunomodulatory activity of PFL was related with concentrations, when the higher the concentration was, the better the immunomodulatory activity was. Which indicated that the immunomodulatory activity of PF was obviously promoted after PF was encapsulated with liposome, and higher encapsulation efficiency played important role in the immunomodulatory activity of PFL. 

In conclusion, PFL was prepared by ethanol injection method, and the preparation conditions of PFL were optimized with RSM. The optimal preparation conditions for PFL was as follows: ratio of lipid to drug (w/w) 9.6 : 1, ratio of soybean phospholipid to cholesterol (w/w) 8.5 : 1, and speed of injection 0.8 mL·min^−1^. Under these conditions, the experimental encapsulation efficiency of PFL was 91.67 ± 0.21%. *In vitro*, PFL not only could significantly promote T lymphocytes proliferation singly or synergistically with PHA, but also could increase the expression levels of IL-2 and IFN-*γ* mRNA, demonstrated the stronger immunoenhancement activity, which provides the theoretical basis for the further experiment *in vivo*.

## Figures and Tables

**Table 1:** Factors and levels of Box-Behnnken experimental design.

Factors	Code	Range and levels		
		−1	0	1
A ( ratio of lipid to drug, w/w)	*X*_1_	5 : 1	10 : 1	15 : 1
B (ratio of soybean phospholipid to cholesterol, w/w)	*X*_2_	6 : 1	8 : 1	10 : 1
C (speed of injection, mL·min^−1^)	*X*_3_	0.3	0.6	1.2

**Table 2:** Response surface Box-Behnken design and experimental encapsulation efficiency (%).

Number	Levels of independent factors			Response EE (%)	
	*X*_1_	*X*_2_	*X*_3_	Practical acquired EE	Predicted acquired EE
1	5 : 1	8 : 1	0.3	76.00	74.99
2	5 : 1	10 : 1	0.6	82.60	82.88
3	15 : 1	6 : 1	0.6	76.90	76.62
4	15 : 1	8 : 1	1.2	72.10	73.11
5	10 : 1	10 : 1	1.2	82.04	81.41
6	15 : 1	10 : 1	0.6	80.15	79.78
7	10 : 1	6 : 1	0.3	76.40	77.03
8	15 : 1	8 : 1	0.3	78.25	77.89
9	10 : 1	6 : 1	1.2	77.00	76.27
10	10 : 1	8 : 1	0.6	92.60	91.30
11	10 : 1	8 : 1	0.6	90.78	91.30
12	10 : 1	10 : 1	0.3	80.02	80.75
13	10 : 1	8 : 1	0.6	91.00	91.30
14	10 : 1	8 : 1	0.6	91.54	91.30
15	5 : 1	6 : 1	0.6	76.80	77.17
16	10 : 1	8 : 1	0.6	90.58	91.30
17	5 : 1	8 : 1	1.2	79.30	79.66

**Table 3:** Estimated regression model of relationship between response variables (EE) and independent variables (*X*_1_,  *X*_2_, and  *X*_3_).

Source	Sun of squares	df	Mean square	*F* value	*P* value prob >*F*
Model	700.32	9	77.81	75.33	<0.0001
A	6.66	1	6.66	6.45	0.0387
B	39.21	1	39.21	37.96	0.0005
C	6.612 × 10^−3^	1	6.612 × 10^−3^	6.402 × 10^−3^	0.9385
AB	1.63	1	1.63	1.57	0.2499
AC	22.33	1	22.33	21.61	0.0023
BC	0.50	1	0.50	0.49	0.5073
*A* ^2^	225.61	1	225.61	218.42	<0.0001
*B* ^2^	99.76	1	99.76	96.58	<0.0001
*C* ^2^	241.12	1	241.12	233.44	<0.0001
Residual	7.23	7	1.03		
Lack of fit	4.60	3	1.53	2.34	0.2150
Pure error	2.63	4	0.66		
Cor total	707.55	16			
	*R* ^2^ = 0.9898		*R*_Adj_^2^ = 0.9766		

**Table 4:** Predicted and experimental values of the responses at optimum conditions.

	Ratio of lipid to drug (w/w)	Ratio of soybean phospholipid to cholesterol (w/w)	Speed of injection/(mL·min^−1^)	EE (%)
Optimum conditions	9.630 : 1	8.470 : 1	0.760	91.59
Modified conditions	9.6 : 1	8.5 : 1	0.8	91.67 ± 0.21

## Abbreviations

PF: Propolis flavonoids
PFL: Propolis flavonoids liposome
RSM: Response surface methodology
PHA: Phytohemagglutinin
ELISA: Enzyme-linked immunosorbent assay
MTT: 3-(4,5-dimethylthiazol-2-yl)-2,5-diphenyltetrazolium bromide
CMF: Calcium and magnesium-free
PBS: Phosphate-buffered saline
DMSO: Dimethyl sulfoxide
EE: Entrapment efficiency
BBD: Box-Behnken design
BL: Blank liposomes
BC: Blank control
IL-2: Interleukin-2
IFN-*γ*: Interferon-*γ*.
